# Potential distribution of *Leptotrombidium scutellare* in Yunnan and Sichuan Provinces, China, and its association with mite-borne disease transmission

**DOI:** 10.1186/s13071-023-05789-y

**Published:** 2023-05-16

**Authors:** Wen-Yu Song, Yan Lv, Peng-Wu Yin, Yi-Yu Yang, Xian-Guo Guo

**Affiliations:** 1 Vector Laboratory, Institute of Pathogens and Vectors, Yunnan Provincial Key Laboratory for Zoonosis Control and Prevention, Dali University, Dali, 671000 Yunnan China; 2 Department of Mathematics and Computer Science, Dali University, Dali, 671003 Yunnan China; 3 State Key Laboratory of Genetic Resources and Evolution, Kunming Institute of Zoology, Chinese Academy of Sciences, Kunming, 650223 Yunnan China

**Keywords:** Climate change, CMIP6, Ectoparasitic mite, Landscape, Prophylactic intervention, Species distribution modeling, Zoonosis

## Abstract

**Background:**

*Leptotrombidium scutellare* is one of the six main vectors of scrub typhus in China and is a putative vector of hemorrhagic fever with renal syndrome (HFRS). This mite constitutes a large portion of the chigger mite community in southwest China. Although empirical data on its distribution are available for several investigated sites, knowledge of the species’ association with human well-being and involvement in the prevalence of mite-borne diseases remains scarce.

**Methods:**

Occurrence data on the chigger mite were obtained from 21 years (2001–2021) of field sampling. Using boosted regression tree (BRT) ecological models based on climate, land cover and elevation variables, we predicted the environmental suitability for *L. scutellare* in Yunnan and Sichuan Provinces. The potential distribution range and shifts in the study area for near-current and future scenarios were mapped and the scale of *L. scutellare* interacting with human activities was evaluated. We tested the explanatory power of the occurrence probability of *L. scutellare* on incidences of mite-borne diseases.

**Results:**

Elevation and climate factors were the most important factors contributing to the prediction of the occurrence pattern of *L. scutellare*. The most suitable habitats for this mite species were mainly concentrated around high-elevation areas, with predictions for the future showing a trend towards a reduction. Human activity was negatively correlated with the environmental suitability of *L. scutellare*. The occurrence probability of *L. scutellare* in Yunnan Province had a strong explanatory power on the epidemic pattern of HFRS but not scrub typhus.

**Conclusions:**

Our results emphasize the exposure risks introduced by *L. scutellare* in the high-elevation areas of southwest China. Climate change may lead to a range contraction of this species towards areas of higher elevation and lessen the associated exposure risk. A comprehensive understanding of the transmission risk requires more surveillance efforts.

**Graphical Abstract:**

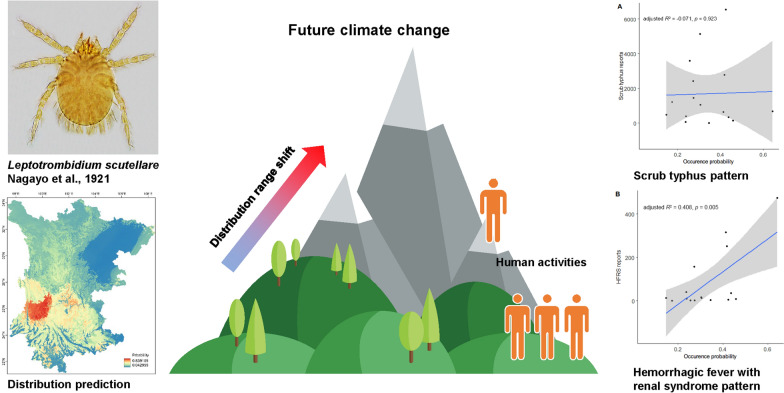

**Supplementary Information:**

The online version contains supplementary material available at 10.1186/s13071-023-05789-y.

## Background

Mite-borne diseases represent a significant proportion of global infectious diseases [1–3]. Reports in recent decades have highlighted an increasing number and range expansion of infectious diseases, such as scrub typhus and hemorrhagic fever with renal syndrome (HFRS) [4–6]. Scrub typhus (also known as tsutsugamushi disease) is caused by the agent *Orientia tsutsugamushi*, a typical mite-borne bacterial pathogen causing zoonotic disease. It is well established that the larvae of some chigger mites (Actinotrichida: Trombiculidae) serve as the exclusive vector of scrub typhus, which can be transmitted from rodents and other wild animals to humans during the parasitic phase through the feeding canal, i.e. a stylostome [7, 8]. HFRS is a viral zoonosis caused by members of the genus* Hantavirus*, and some chigger species are suspected to be possible vectors of the disease [9].

Six chigger species belonging to the genus *Leptotrombidium*, including* L. scutellare* (Nagayo et al., 1921), *L. deliense* (Walch, 1922), *L. rubellum* Wang and Liao, 1984, *L. wenense* Wu, Wen, Yang and Wu, 1982, *L. insulare* Wei, Wang and Tong, 1989 and *L. sialkotense* Vercammen-Grandjean and Langston, 1976, have been confirmed as the main vectors of scrub typhus in mainland China [10]. These mite species are known to occur in Yunnan Province and the adjacent areas, with *L. scutellare* and *L. deliense* being the dominant species [11–14]. *Leptotrobidium scutellare* is considered to be one of the possible vectors of Hantavirus, an emerging zoonotic disease [3, 15–17]. No longer than a decade ago, *L. scutellare* was mostly reported in northern China where it was considered to be the main vector of scrub typhus, occurring at latitudes higher than 31° N [8, 18]. However, field observations in the last decade demonstrated an extensive distribution of *L. scutellare* in the provinces of southwest China [19, 20], effectively challenging the notion that *L. deliense* was the main vector of scrub typhus in the region [18]. The outcomes of these observations prompted more efforts in studying the distribution and epidemic relevance of *L. scutellare*. A better understanding of the distribution of *L. scutellare* may provide knowledge contribution to the prevention of associated diseases and benefit public health interventions.

Recently, considerable efforts have been made to better understand the distribution of *L. scutellare* and its role in disease transmission. It has been reported that the presence of *L. scutellare* and the epidemic incidence of scrub typhus were identical in South Korea [21]. These results suggest that changes in the distribution of the vector may affect the corresponding epidemic patterns. A recent study outlined the potential distribution range under simulated future environments of the two most widely distributed vector chiggers *L. deliense* and *L. scutellare* in mainland China [22]. Another study mapping the distributions of blood-sucking mites and mite-borne agents also indicated that *L. scutellare* harbors the highest variety of pathogens [23], which is turn increases the exposure risk to relevant diseases for the human population where the vector species occurs.

Although these earlier studies contributed significant data towards improving our knowledge of the spatial and temporal dynamics of chigger vectors, they relied on the presence data of the mites with coordinates extracted from center points of the corresponding administrative areas, i.e., the counties [24]. Thus, these projections may underestimate the heterogeneity of environmental suitability for the species within the areas, especially when field observations have shown that mites such as *L. scutellare* showed specialized habitat preference, i.e. uncultivated habitats at mid to high elevations [12, 25]. Taking environmental differences within the sampled areas into account when evaluating the distribution may improve the performance of ecological models. Such an attempt is realistic only when the collection site is accurately documented. So far, the occurrence of *L. scutellare* in southwest China has been reported at the county level [19, 24]. The suitable area and potential distribution of *L. scutellare* in southwest China have not been exhaustively discussed, and the interactions between *L. scutellare* and anthropic activities, such as cropping, logging or tourism, are unexplored. Also, the epidemic role of *L. scutellare* in mite-borne disease prevalence in southwest China is unclear.

**Fig. 1:**
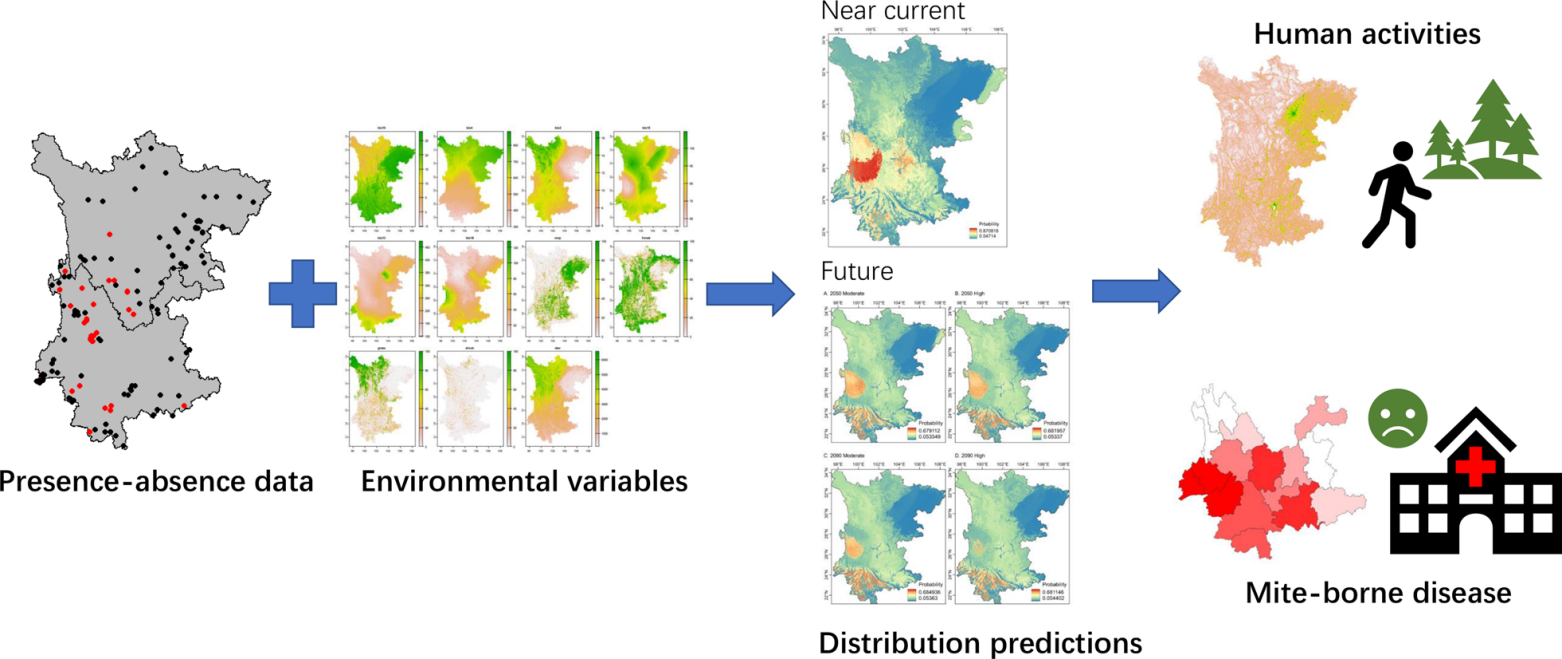
Workflow of the present study

Based on the occurrence data of chigger mites collected continuously in 21 years of field investigations, we explored three main questions: (i) what is the potential distribution range of *L. scutellare* in the study area under near current and future climate scenarios; (ii) how much does the distribution range of *L. scutellare* overlap with human activities; and (iii) does the potential distribution range of *L. scutellare* explain the prevalence of scrub typhus and HFRS in the study region (see the workflow in Fig. 1). Through these efforts, we aimed to evaluate the exposure risk to scrub typhus and HFRS from *L. scutellare* in Yunnan and Sichuan Provinces on both spatial and temporal scales.

## Methods

### Study area

**Fig. 2:**
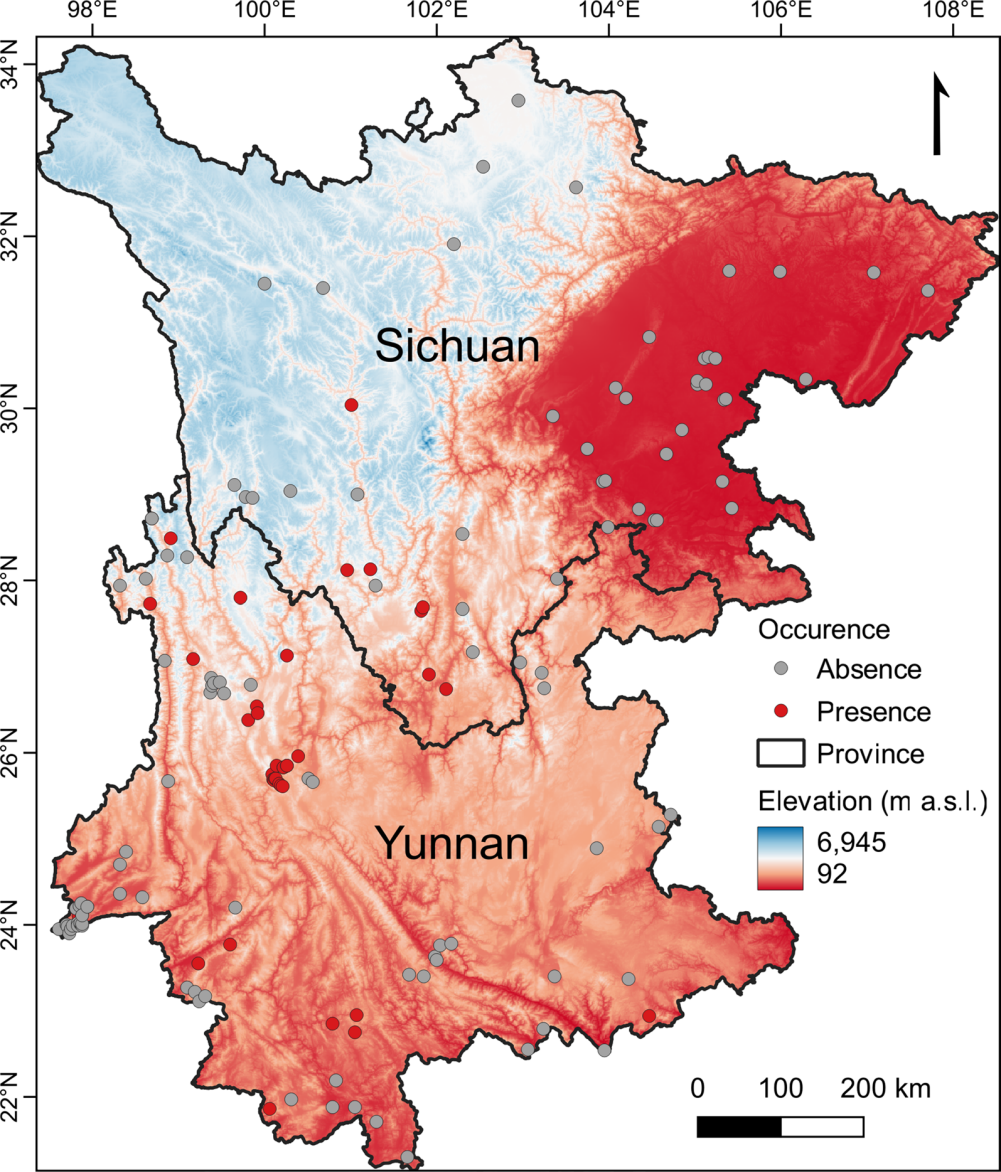
Map showing the topography of the study area and sampling points investigated for the presence/absence of *Leptotrombidium scutellare*

The study was conducted in Yunnan and Sichuan Provinces, two adjacent provinces in southwest China. The uplift of the Qinghai-Tibet Plateau resulted in the formation of various mountain ranges in this region and, consequently, an extremely high heterogeneity in its topography and climate (Fig. 2). Elevation generally increases from the southeast to northwest, with the mean annual temperature decreasing in the same direction. Moisture delivered by the seasonal monsoon causes a decline in precipitation from west to east. Based on these variations, the study area covers almost all types of existing climates worldwide, from wet or dry tropical to alpine tundra [26]. The western part of the study region is dominated by a spectacular mountain-valley system with vast elevational differences (between 76 and 7556 m a.s.l.), which is known as the Longitudinal Range and Gorge Region of southwest China [27]. Various vegetation types are distributed among this mountain-valley system, including humid evergreen broad-leaved forest and semi-arid bushes along the valleys, mixed broadleaf-conifer forest and dark coniferous forest on mid- and high-elevation slopes, and meadows and tundra in alpine areas. Eastern Sichuan is marked by a low-elevation basin (250–700 m a.s.l.) surrounded by mountains (1000–3000 m a.s.l.). This basin occupies approximately 33% of the area of Sichuan Province and is mostly covered by subtropical evergreen broad-leaved forest except for the areas with human modifications. The western Sichuan area is formed by a vast plateau with an average elevation of > 4000 m a.s.l., which is predominantly covered by oak bushes, meadows and scree. The dark coniferous forest can also be seen in a few valleys in this area. Eastern and middle Yunnan Province is generally dominated by mountainous highlands (approx. 2000 m a.s.l.). Mixed broadleaf-conifer forests are widely distributed in these highlands. Low-elevation plains and hills predominantly constitute the topography in southern Yunnan (< 1000 m a.s.l.), which is generally covered by tropical rainforest and humid evergreen broad-leaved forest. Human settlements and croplands are spread over the valleys, plateaus and low-elevation plains in the study region.

### Field sampling and occurrence data of *L. scutellare*

Field sampling was conducted in 149 sites across 69 counties in Yunnan and Sichuan Provinces from 2001 to 2021. Animal hosts were collected in cage traps (18 × 12 × 9 cm; Guixi Mousetrap Apparatus Factory, Guixi, Jiangxi, China) baited with fresh peanuts, corn or other standard baits [28]. Chiggers were collected from the body surface of each host separately, following a standardized protocol [29]. The collected chiggers were mounted on glass slides in Hoyer’s medium and identified under an optical microscope. The specimen vouchers are deposited in the specimen repository of the Institute of Pathogens and Vectors, Dali University, China. A total of 178,942 chiggers were collected and subsequently identified as 302 species, with 1053 individuals of *L. scutellare* determined to be present in 40 of the 149 investigated sampling sites (Fig. 2).

### Environmental variables

The distribution range and population persistence of chigger mites have been found to be determined by climate factors and landscape [7, 12, 29, 30]. However, there is no consensus on which climate factor is the most important. To estimate the species-environment relationship and map the potential distribution of *L. scutellare*, layers of 19 bioclimatic variables relating to temperature and precipitation measures spanning 1970–2000 were downloaded from the Worldclim 2.1 web server (for full description for the 19 bio-climate variables, see https://worldclim.org/data/index.html). The ‘select07’ function in the ‘mecofun’ package was then used to identify the most important climate variables and reduce multi-collinearity [31]. A linear regression model was imposed on these variables to evaluate their explanatory power (adjusted *R*^2^) on the occurrence of *L. scutellare*.

Land cover types (e.g. type of vegetation) were considered a predominant determinant of chigger occurrences in previous studies [12, 32, 33]. We obtained land cover data indexing cropland, shrubland, grassland or forest from a global land use dataset (1-km resolution) [34]. Multiple ecological factors can vary dramatically along the elevational gradient [35, 36]. For *L. scutellare*, field observation data suggested that elevations between 2000 and 2500 m a.s.l. were the preferred range [12]. Thus, we included elevation as a synthesis variable representing any undefined factors. We used the 30-s resolution elevational layer from the Shuttle Radar Topography Mission downloaded from the Worldclim 2.1 web server.

For future scenarios, we incorporated the projections based on the Coupled Model Intercomparison Project Phase 6 (CMIP6) [37] for climate variables and the Cellular Automata models for land cover change [34]. The CMIP6 replaced the previously used Representative Concentration Pathways (RCPs) with the updated Shared Socio-economic Pathways (SSPs). Four SSPs scenarios (SSP1-2.6, SSP2-4.5, SSP4-6.0, and SSP5-8.5) have levels of radiative forcing that correspond with the RCPs (RCP2.6, RCP4.5, RCP6.0 and RCP8.5) [38]. The BCC-CSM2-MR climate model (a high-resolution climate system model released by the Beijing Climate Center [BCC]) has been found to outperform other general circulation models in certain parameters [39, 40]. Thus, SSP2-4.5 and SSP5-8.5 derived from the model were selected to simulate moderate and high greenhouse gas concentration scenarios, respectively. The layers containing future climate projections (2050, as a climatology computed from 2041–2060; 2090, as a climatology computed from 2081–2100) were also obtained from the Worldclim 2.1 web server. The 30-s resolution land cover layers of the years 2050 and 2090 under the RCP4.5 and RCP8.5 scenarios were obtained from Li et al. [34]. Ultimately, elevation and bioclimatic layers comprising two future periods (2050 and 2090) under moderate (SSP2-4.5 or RCP4.5) and high (SSP5-8.5 or RCP8.5) greenhouse scenarios were used for future distribution projection. All eco-geographic layers were cropped by the extent of Yunnan and Sichuan Provinces, respectively, and resampled to 30 s when necessary for further assessments.

### Model fitting and distribution projection

Environmental variables were extracted from the selected layers of the near-current period by the presence-absence points with the ‘extract’ function provided by the ‘terra’ package [41]. We fitted a boosted regression tree (BRT) ecological model based on a case-control research strategy for species distribution modeling [42]. The data points of the investigated sites were assigned as “cases” when *L. scutellare* is present (1) and “controls” when the species is absent (0). Of all data points, 75% were extracted randomly as a training set, and the remaining 25% were treated as a test set [23]. The training set was used to build a BRT model and to acquire the relative contributions of the variables using the ‘gbm.step’ function provided in the ‘dismo’ package [43]. The model was run with a tree complexity of 5, a learning rate of 0.005 and a bagging fraction of 75% based on the satisfactory performance in previous practice runs [23, 44]. Tenfold cross-validation was adopted to produce the receiver-operating characteristic (ROC) curves. The model performance and predicted power were evaluated by the areas under the curve (AUC). The AUC of the fitted model was derived with the ‘evalSDM’ function available in the ‘mecofun’ package using the test set for validation [31]. The training set extraction, BRT model fitting and predictive performance evaluation were repeated 100 times. We averaged the values provided by the repetitions to increase the robustness of the modeling performance [22].

For each focused scenario and period, the occurrence probability was predicted based on the BRT model products of 100 repetitions with the ‘predict.gbm’ function available in the ‘gbm’ package [45]. The outputs were also averaged for geographical mapping. The ‘evalSDM’ function also returns a cut-off value (threshold) based on the maximum sensitivity+specificity along the ROC curve that can be used to translate the predicted probabilities of *L. scutellare* into binary levels [31]. Pixels with probabilities beyond the threshold were considered suitable (1), while those below the threshold were considered unsuitable (0) for *L. scutellare* to map the binarizing occurrence patterns.

### Relating occurrence probability with human activities and mite-borne diseases

The Human Footprint index shows the gradient of human activities [46]. It is a weighted sum of maps of where people live (population density) and the accessibility to different areas (e.g. infrastructure). We downloaded the layer mapping the Human Footprint for the year 2020, released by the Wildlife Conservation Society (https://wcshumanfootprint.org/data-access), and then re-sampled (bilinear interpolation) the Human Footprint layer to 30 s. We calculated the Pearson’s correlation coefficients between the Human Footprint index and the occurrence probability of *L. scutellare* under near-current and future projections with the ‘raster.cor.matrix’ function provided in the ‘ENMTools’ package [47].

Finally, we fitted linear regression models to evaluate the explanatory power of the occurrence probability of *L. scutellare* for the mite-borne disease frequencies in recent decades. Because case reports for the mite-borne disease were not publicly available for Sichuan Province, in the present study we only analyzed data on Yunnan Province. An area with more suitable habitats for *L. scutellare* should have a higher occurrence probability and, consequently, a higher infection rate for mite-borne diseases. We first averaged the predicted probability of *L. scutellare* at the city level and then derived the epidemiological patterns of mite-borne diseases from published literature. We obtained the case reports of scrub typhus in Yunnan Province during the period 2006–2017 [5]. The case reports of HFRS in Yunnan Province had been aggregated by the periods 1976–2012 [48] and 2014–2015 [49]. The number of case reports was used as a response variable, and the probability of *L. scutellare* was used as an explanatory factor when fitting the linear regression models. The occurrence data were primarily organized in Microsoft Excel (Microsoft Corp., Redmond, WA, USA), while the eco-geographic layers and statistical analyses were processed in the R 4.1.0 environment [50]. The output maps were assembled in QGIS 3.18 [51].

## Results

### Model ensembles and predictive performance

During the field investigation, *L. scutellare* was collected from 34 species of small mammals, with *Eothenomys miletus*, *Apodemus chevrieri*, *Tupaia belangeri* and *Neotetracus sinensis* being the dominant hosts. The average prevalence of *L. scutellare* in sites where the species occurred was 12.1% (*n* = 1053/8728). The variable selection process retained six of the initial set of 19 bioclimatic variables while maintaining most of the explanatory power for *L. scutellare* occurrence after removal of highly correlated variables. These six variables were temperature mean diurnal range, temperature seasonality, mean temperature of warmest quarter, precipitation of wettest month, precipitation seasonality and precipitation of coldest quarter. Linear regression showed that these six climate variables together explained 18.9% of the presence-absence pattern of *L. scutellare* in the study area.

The average (± standard deviation [SD]) model AUC was 0.85 ± 0.07, indicating that the ensembled BRT model had a high predictive performance. Elevation (mean ± SD, 24.4 ± 5.7%) yielded the highest contribution to predicting environmental suitability (Table 1), followed by temperature seasonality (22.1 ± 5.5%) and precipitation seasonality (19.1 ± 7.3%). Shrubland contributed the least (0.3 ± 0.4%) of the 11 variables. Model performance evaluation returned a cut-off value of 0.35 ± 0.18 to determine the binary suitability for *L. scutellare* in the study area. The marginal effects of the 11 environmental variables are visualized in Additional file 1: Figure S1.

**Table 1:** Relative contributions of the 11 environmental variables used to predict the distribution of *Leptotrombidium scutellare* ensembled by the boosted regression tree model

Variable	Relative contribution
Elevation	24.36 ± 5.70
Temperature Seasonality	22.05 ± 5.52
Precipitation Seasonality	19.09 ± 7.29
Mean Temperature of Warmest Quarter	9.52 ± 4.10
Temperature Mean Diurnal Range	5.74 ± 2.71
Precipitation of Coldest Quarter	5.52 ± 2.15
Precipitation of Wettest Month	5.01 ± 1.99
Cropland	3.23 ± 1.36
Forest	3.22 ± 1.73
Grassland	1.89 ± 1.40
Shrubland	0.33 ± 0.40

Values in table are expressed as the mean ± standard deviation (SD)

### Distribution range shifts

**Fig. 3:**
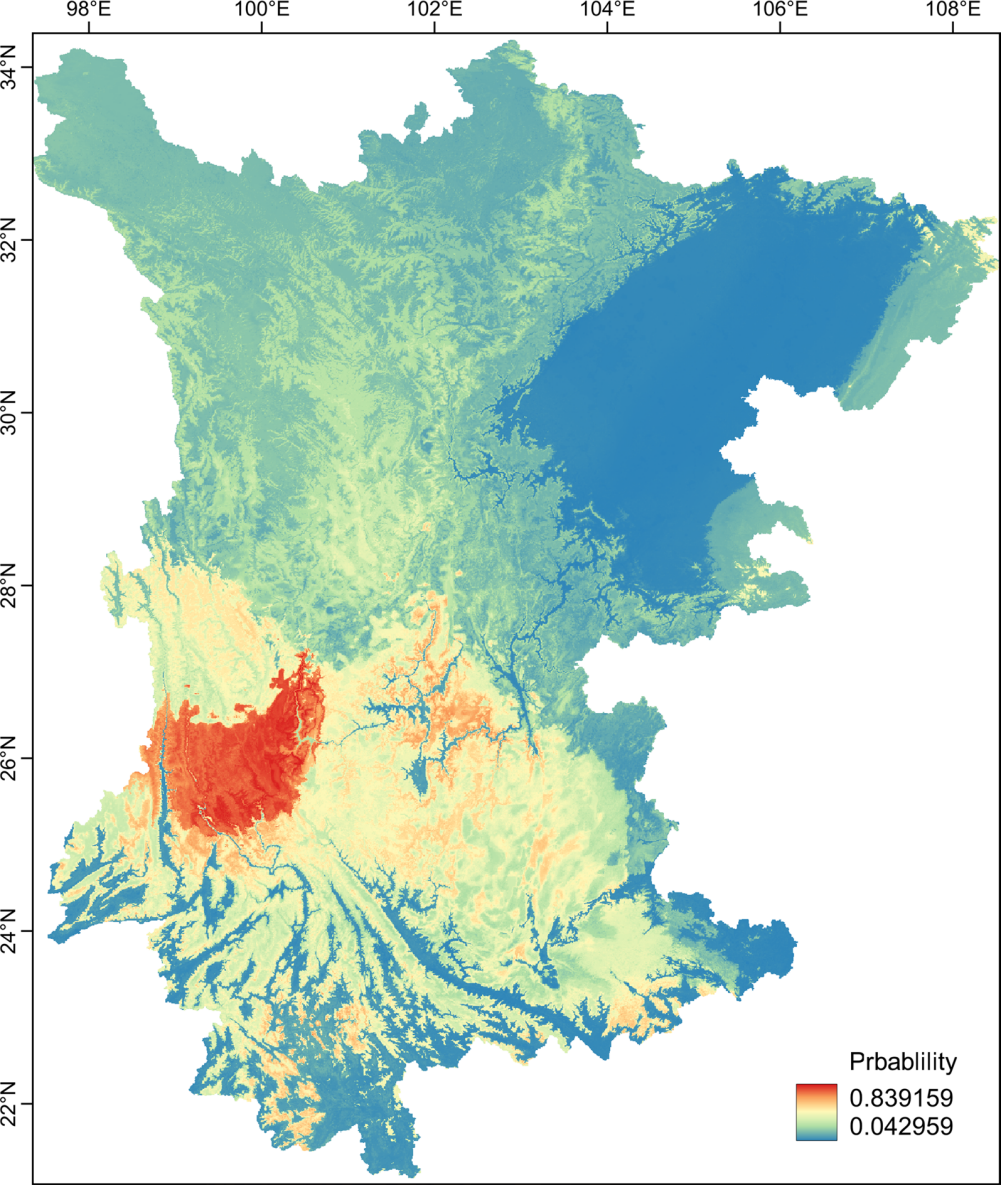
Potential suitable areas for *Leptotrombidium scutellare* occurrence in Yunnan and Sichuan, China in the near-current period

**Fig. 4:**
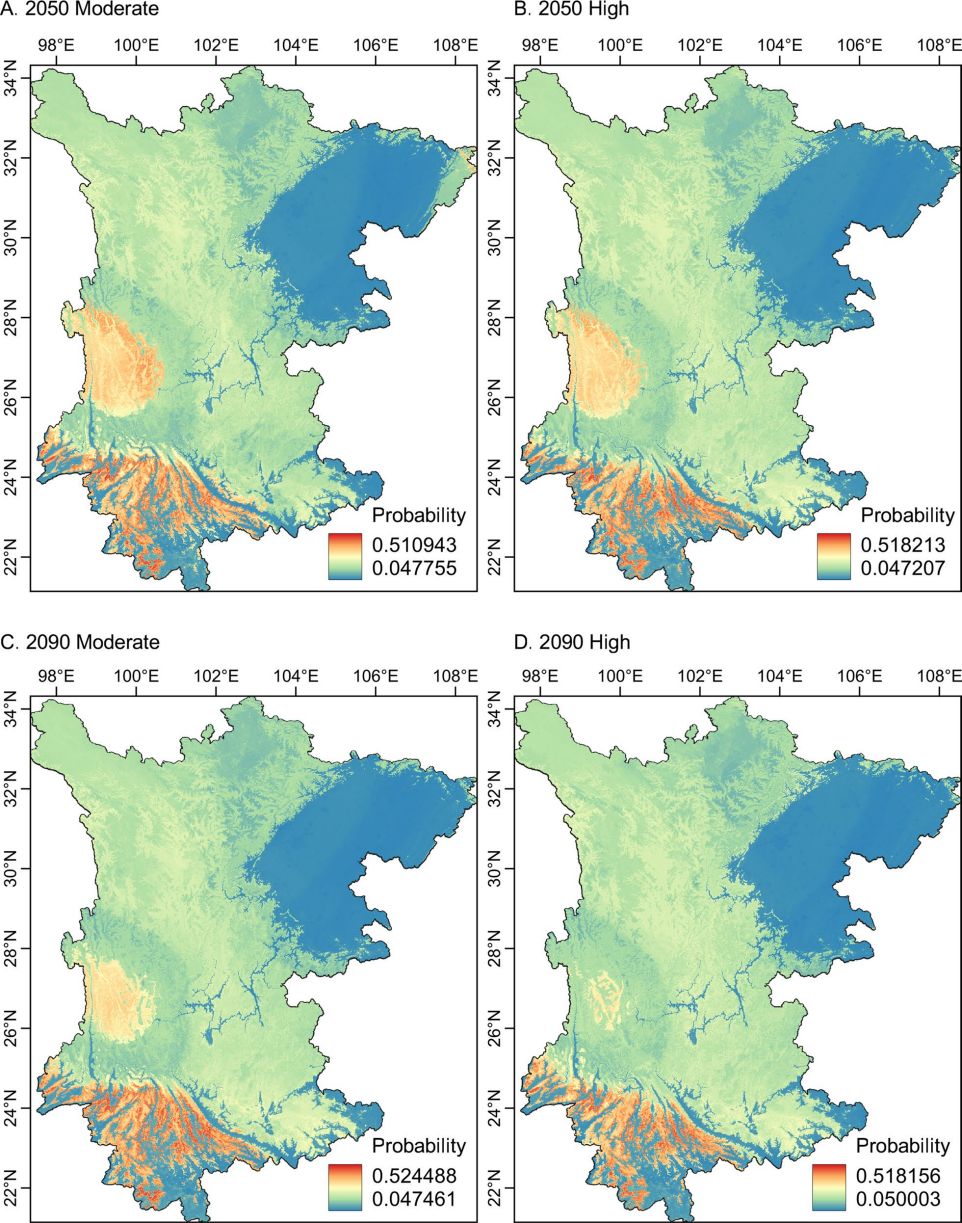
Projections of the suitable areas for *Leptotrombidium scutellare* occurrence in two future periods (2050 and 2090) under moderate (**a**,** c**) and high (**b**,** d**) greenhouse gas concentrations

Geographical projection suggested that the suitable areas for *L. scutellare* were mostly spread over the western Yunnan plateau and southwest Yunnan mountain ridges (Fig. 3). Compared with these areas, the plateau in western Sichuan was less suitable. The Sichuan basin and the southern Yunnan lowlands were consistently recognized as unsuitable in the near-current and future periods (Fig. 4). The predicted areas suitable for *L. scutellare* were 214,272.40 km^2^ in the near-current period (Additional file 1: Figure S2). In the future simulations of moderate greenhouse gas concentrations, the suitable areas were reduced to 55,916.57 km^2^ in 2050 and 52,064.95 km^2^ in 2090. Under high greenhouse gas concentrations, the suitable areas in the study region were further reduced to 51,304.36 km^2^ in 2050 and 41,217.22 km^2^ in 2090, respectively (Additional file 1: Figure S3).

### Correlations between *L. scutellare* and exposure risks to mite-borne diseases

The correlations between the Human Footprints index and the predicted occurrence probabilities of *L. scutellare* in the near-current and future periods were negative (Table 2), indicating the dispreference of habitats with intensive human activities. The correlation coefficient was weakly negative in the near-current period (Pearson’s *r* = − 0.159). The negative correlations between the occurrence probabilities of *L. scutellare* and anthropic activities tended to be enhanced over time as the greenhouse effect increased (Table 2).

**Table 2:** Pearson’s correlation coefficients between Human Footprints and the projected distribution patterns of *Leptotrombidium scutellare* in different periods and greenhouse gas concentration scenarios

Periods	Human Footprints index	Near-current period	Future periods			
			2050 moderate greenhouse gas scenario	2090 moderate greenhouse gas scenario	2050 high greenhouse gas scenario	2090 high greenhouse gas scenario
Near-current	− 0.159	1				
2050 moderate greenhouse gas scenario	− 0.332	0.700	1			
2090 moderate greenhouse gas scenario	− 0.300	0.655	0.933	1		
2050 high greenhouse gas scenario	− 0.332	0.698	0.947	0.943	1	
2090 high greenhouse gas scenario	− 0.326	0.610	0.876	0.932	0.908	1

**Fig. 5:**
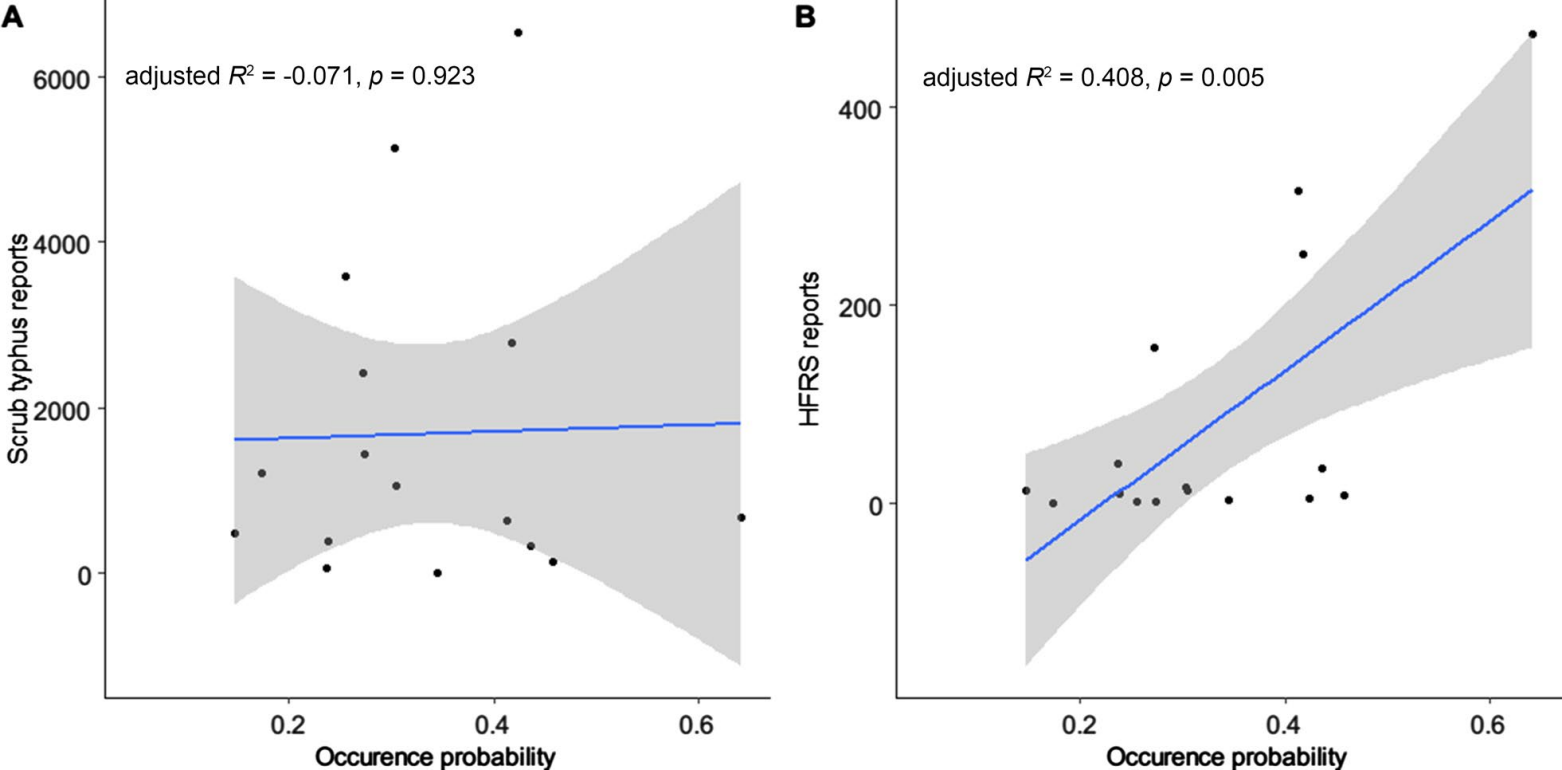
Linear correlations between the potential occurrence probabilities of *Leptotrombidium scutellare* and mite-borne disease incidences in the cities of Yunnan Province. The blue line shows the linear regression line and the gray area is the 95% confidence interval. Black dots mark the cities used to fit the regression model. HFRS, Hemorrhagic fever with renal syndrome

In the near-current period, the cities with the highest average occurrence probability of *L. scutellare* were Panzhihua (0.43) in Sichuan Province and Dali (0.64) in Yunnan Province, while Suining (0.05) and Zhaotong (0.15) reach the lowest average occurrence probability in Sichuan and Yunnan Provinces, respectively (Additional file 1: Table S1). The linear regression models showed that the occurrence probabilities of *L. scutellare* in Yunnan Province had high explanatory power for the incidence of HFRS (adjusted *R*^2^ = 0.41, *F*[14] = 11.32, *P* = 0.005) but not for the incidence of scrub typhus (adjusted *R*^2^ = − 0.07, *F*[14] = 0.01, *P* = 0.923) (Fig. 5).

## Discussion

### Potential distribution range in near-current and future periods

In the present study, the results of the BRT models showed that elevation and climate factors explained most of the occurrence pattern of *L. scutellare* (> 91%), with elevation contributing the strongest to the prediction of the occurrence pattern. This result is in contrast with those of a recent study in which it was found that elevation contributed little (no more than 5%) to the species in their models [22]. This difference can be explained by the fact that our point data contains more accurate information on the presence of *L. scutellare* along the elevational gradients. The mountainous areas of southwest China are marked by a vast variation in elevation [27], with corresponding variations in environmental factors, such as temperature, water resources, vegetation cover and soil chemistry [36]. Such variations in elevation have been found to impact the distribution of small mammal hosts [52] and their ectoparasitic species [25]. Our results are in line with the previous finding that *L. scutellare* prefers mid- to high-elevation habitats [12], further highlighting the important influence of topography in the distribution of the species.

Climate variables constituted a considerable portion of the contribution to predicting the occurrence pattern. Findings from previous studies suggested a negative correlation between temperature and *L. scutellare* presence [53, 54]. *Leptotrombidium scutellare* is known to be widely distributed in northern China, which is colder in winter compared to the southern regions of the country [24, 55]. The species is also known to be more active during the cold seasons and to act as the main vector of scrub typhus in southern China during winter [18, 56]. Thus, an area with a cold winter in low latitudes may represent optimization of the environmental suitability for *L. scutellare*. The high contribution of precipitation seasonality to predicting the occurrence pattern of *L. scutellare* may be attributed to the life history traits as it has been noted that rainfall heavily influences the reproduction and growth of this chigger species, as well as that of many other species [57]. Overall, the results of the present study support the view that the occurrence of *L. scutellare* is highly sensitive to climatic and topographic heterogeneity [7, 12].

Land cover variables explained only a small portion of the occurrence pattern (< 9%). It has been suggested that there is no consistent effect of land covers such as woodlands, scrublands, hedgerows and grasslands on the abundance of pests and pathogens and that land cover has a small effect compared to climate [58]. Our results support the idea that climate generally outweighs land covers in determining the occurrence of *L. scutellare*. Previous studies supported a higher prevalence of chiggers in densely vegetated lands [2, 32]. Our results showed that croplands and forests are generally more suitable for *L. scutellare* than shrublands and grasslands. There are a number of possible explanations for this result. Firstly, forest and cropland constitute most of the land cover area in the study region, with shrublands and grasslands appearing only as small patches amidst them. Secondly, the local people in southwest China have developed unique cropping methods due to the mountainous terrain, and these differ from those used in flat plains; for example, people in southwest China developed the famous terrace farming to cultivate crops alongside mountainous forest habitats. This cropping method may create a transitional zone that is favored by small mammal hosts and their ectoparasitic chiggers. A similar positive correlation has been reported between scrub typhus incidence and Taiwan's mosaic landscapes of cropland and forest [59].

The geographical projection showed that under the near-current period scenario, plateau and high-elevation mountains are generally more suitable than plains and valleys. These results suggest that people living in mountainous areas likely have higher transmission risks related to *L. scutellare*. The occurrence probabilities of suitable areas for *L. scutellare* in Yunnan and Sichuan Provinces decreased with increasing greenhouse effect, possibly because this species prefers a colder environment compared to other vector mite species in Yunnan Province [60, 61]. Therefore, under future temperature increases, the range of *L. scutellare* may contract to high-elevation mountains. Such a tendency was also reported in other mountain-dwelling species, especially those adapted to cold temperatures and high-elevation habitats, for example, the Himalayan coldwater specialist snow trout (*Schizothorax richardsonii*) [62] and herbages restricted to alpine habitats [63]. However, our results are in contrast with a previous projection in the distribution tendency. A recent study found that both *L. scutellare* and *L. deliense* had broader suitability ranges in the high RCP scenario, suggesting a future northward expansion of geographic distribution [22]. This discrepancy may be attributed to their occurrence points not representing the preferable elevation of *L. scutellare* [24]. *Leptotrombidium scutellare* and *L. deliense* can co-exist in a given area yet occupy different elevations and landscapes [12, 19]. Thus, including accurate environmental information on the collection site in the ecological model may improve our understanding of environmental suitability for the species.

Despite the difference in directions, our results are in agreement with previous studies in regarding that the greenhouse effect may optimize habitat suitability in a colder environment, i.e. high elevation or high latitude. Combined, our results of the distribution mapping study indicate that increased temperature may drive the upward or northward shifts for *L. scutellare* to pursue a more suitable habitat [64, 65]. Moreover, although the potential distribution range of *L. scutellare* may be reduced in the future, the temperature rise may expand the suitable habitats for other vector mites, such as *L. deliense* and *L. rubellum*, which favor warmer conditions. For example, opposite responses to climate change were detected between two small mammal hosts in the mountains of southwest China [66].

### Evaluation of the risks introduced by *L. scutellare*

The results of the present study indicate a low correlation between the potential distribution patterns of *L. scutellare* and the Human Footprint index in the near-current period. This result is due to the preference of this species for a mountainous uncultivated landscape and because it is primarily parasitic on hosts that live in the wilderness [12, 56]. Studies in South Korea [33] and Thailand [32] suggested that human disturbance and urbanization may mitigate the risk of rickettsioses transmission because the vector chiggers generally prefer habitats less modified by people. Nevertheless, the increasing occurrence probability of *L. scutellare* may increase the infection risk for people living in high-elevation areas. These people may have a higher opportunity to engage the vector due to their residences and activity patterns being close to the suitable habitats of *L. scutellare*. On the other hand, *L. scutellare* was found to peak in population size in cold seasons [7] and was noted to act as the main vector of autumn-winter-type scrub typhus [67]. The forest fire-safe periods in winter and spring, which forbid most human entry into the mountainous areas in Yunnan and Sichuan Provinces, may help to reduce the exposure risk. The interactions between *L. scutellare* and human activities were expected to decline gradually under future scenarios due to the contraction of suitable habitats. It is worth noting that these results were valid only in a certain context because they were built on the assumption that the intensity of anthropic activities remains constant for the following decades. However, the contraction of *L. scutellare* range does not necessarily correspond to a low exposure risk [44] because other vectors that coexist in the study region may expand their distribution under climate change scenarios [22, 66]. Therefore, a study on a multi-species level focusing on the study region is needed to improve the evaluation of exposure risk to mite-borne diseases.

Our results showed that the occurrence probability of *L. scutellare* did not explain the incidence of scrub typhus in Yunnan Province. As such, our findings are not in agreement with those of a study in South Korea that suggested a congruent pattern between the mapped distribution of *L. scutellare* and the incidence of scrub typhus [21]. A plausible explanation for our results is that the distribution of *L. scutellare* was mostly restricted to less urbanized areas in southwest China. This leads to a confined influence of the species on the majority of the human population. The incidences of scrub typhus were mostly reported in cities representative of the low-elevation areas of southern Yunnan [5]. These cities were also likely favored by *L. deliense*—another major *O. tsustugamushi* vector adapted to warmth and low elevation [61]. However, high suitability for *L. scutellare* in the plateaus of Yunnan Province may still drive the increasing trend of scrub typhus in these areas, such as in Kunming and Dali [5, 68].

We found strong support for a positive correlation between the occurrence probability of *L. scutellare* and the incidence of HFRS in Yunnan Province. This result highlights the potential for *L. scutellare* playing a considerable role in HFRS transmission. The hantavirus has been segregated from multiple small mammal hosts in the wild, including members of the genera *Apodemus* and *Eothenomys* [69, 70]. *Leptotrombidium scutellare* has been reported to be a dominant ectoparasitic species on these hosts [12]. It is worth noting that to date the epidemiological evidence of transmission of HFRS through *L. scutellare* remained unsatisfactory [56]. Thus, more research may be needed to establish a straightforward association between *L. scutellare* and HFRS.

Our analysis encountered several unavoidable limitations. Many areas in the study region have not been adequately investigated. Thus, more surveillance efforts in the future are required to increase our knowledge of the actual distribution of *L. scutellare* and other potential vector mites. The predictions of exposure risk were based on the human activity data available for the contemporary period. This interaction may be exacerbated or mitigated depending on the forest management policy, the progress of urbanization and the development of farming, tourism and infrastructure construction. We could not address the correlations between the occurrence of the chigger with relevant diseases such as scrub typhus and HFRS in Sichuan Province. Further study should target such an issue to better understand the role of potential vectors underlying the epidemic patterns.

## Conclusion

Our results showed that people living in high-elevation areas are likely exposed to mite-borne diseases transmitted by *L. scutellare*. Future climate change is expected to reduce the suitable areas for *L. scutellare* in Yunnan and Sichuan Provinces. Such range shifts may mitigate the range overlap between humans and *L. scutellare.* We found that the occurrence probability of the species yielded contrasting associations between different mite-borne diseases, emphasizing the necessity of more study to understand the underlying epidemiological mechanisms. Overall, our findings provide vital insight into prophylactic interventions for improving public health in the targeted region.

## Supplementary Information

Additional file 1: Figure S1. Marginal effect curves of the 11 environmental variablesover 100 boosted regression treemodels. Red lines indicate the average effect curves and the gray zone marks the predicted 95% confidence interval. Figure S2. Binarizing distribution range of L. scutellare in the near-current period. Figure S3. Binarizing distribution range of L. scutellare in the future periods. a Moderate greenhouse effect in 2050; b high greenhouse effect in 2050, c moderate greenhouse effect in 2090, d high greenhouse effect in 2090. Table S1. Average occurrence probability of L. scutellare in the cities of Yunnan and Sichuan Provinces, and the numbers of case reports of HFRS and Scrub typhus in Yunnan.

## Data Availability

The original contributions presented in the study are included in the article/Supplementary material, further inquiries can be directed to the corresponding author.

## Abbreviations

BRT: Boosted regression tree
CMIP6: Coupled Model Intercomparison Project Phase 6
HFRS: Hemorrhagic fever with renal syndrome
