# Supplementary material for: Potential distribution of Leptotrombidium scutellare in Yunnan and Sichuan Provinces, China, and its association with mite-borne disease transmission
Source: Parasit Vectors. 2023 May 16;16:164. doi: 10.1186/s13071-023-05789-y (PMC10190071; doi:10.1186/s13071-023-05789-y)
Supplement: Supplementary file 1 — Additional file 1: Figure S1. Marginal effect curves of the 11 environmental variablesover 100 boosted regression treemodels. Red lines indicate the average effect curves and the gray zone marks the predicted 95% confidence interval. Figure S2. Binarizing distribution range of L. scutellare in the near-current period. Figure S3. Binarizing distribution range of L. scutellare in the future periods. a Moderate greenhouse effect in 2050; b high greenhouse effect in 2050, c moderate greenhouse effect in 2090, d high greenhouse effect in 2090. Table S1. Average occurrence probability of L. scutellare in the cities of Yunnan and Sichuan Provinces, and the numbers of case reports of HFRS and Scrub typhus in Yunnan. [file 13071_2023_5789_MOESM1_ESM.docx]

**Potential distribution of *Leptotrombidium scutellare* in Yunnan and Sichuan Provinces of China, and its association with mite-borne diseases transmission**

**Wen-Yu Song ^1,3^, Yan Lv ^1^, Peng-Wu Yin ^1^, Yi-Yu Yang ^2^, Xian-Guo Guo ^1^***

1. Vector Laboratory, Institute of Pathogens and Vectors, Yunnan Provincial Key Laboratory for Zoonosis Control and Prevention, Dali University, Dali, Yunnan 671000, China

2. Department of Mathematics and Computer Science, Dali University, Dali, Yunnan 671003, China

3. State Key Laboratory of Genetic Resources and Evolution, Kunming Institute of Zoology, Chinese Academy of Sciences, Kunming, Yunnan 650223, China

*Correspondence: xgguo2002@163.com

**Supplementary information**

**Additional file 1: Figure S1** Marginal effect curves of the 11 environmental variables (ordered by the relative contribution) over 100 boosted regression tree (BRT) models. Red lines indicate the average effect curves and the gray zone marks the predicted 95% confidence interval


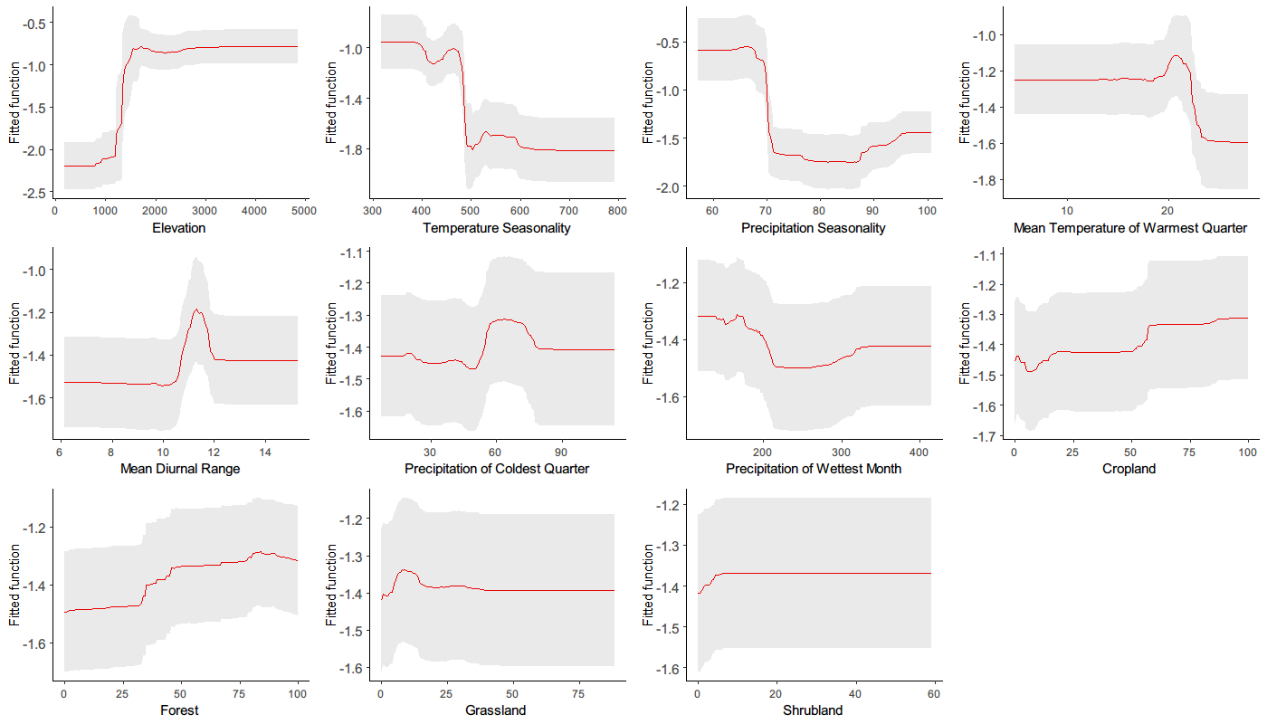


**Additional file 1: Figure S2** Binarizing distribution range of *L. scutellare* in the near current period


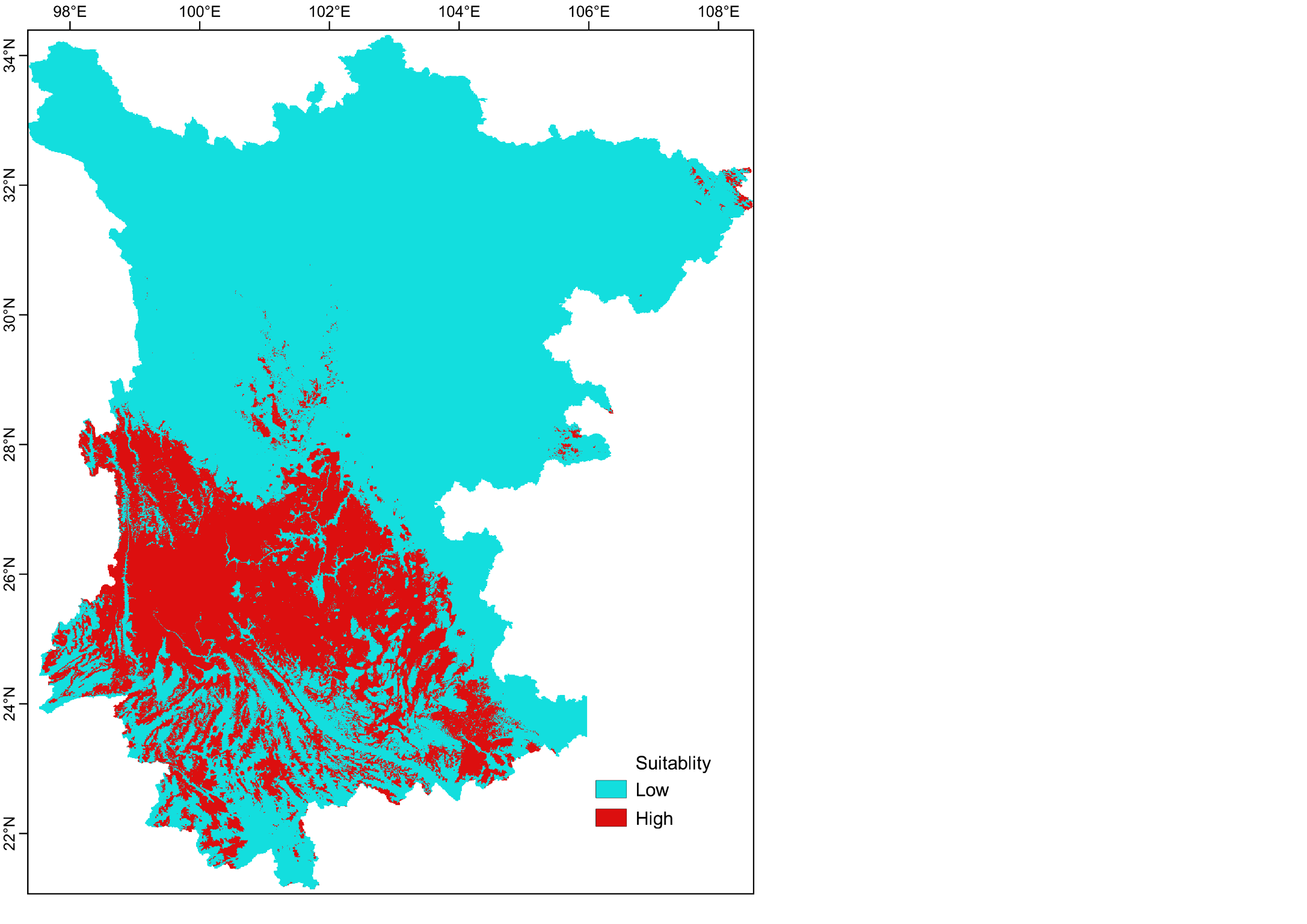


**Additional file 1: Figure S3** Binarizing distribution range of *L. scutellare* in the future periods. A) Moderate greenhouse effect in the year 2050; B) High greenhouse effect in the year 2050; C) Moderate greenhouse effect in the year 2090; D) High greenhouse effect in the year 2090


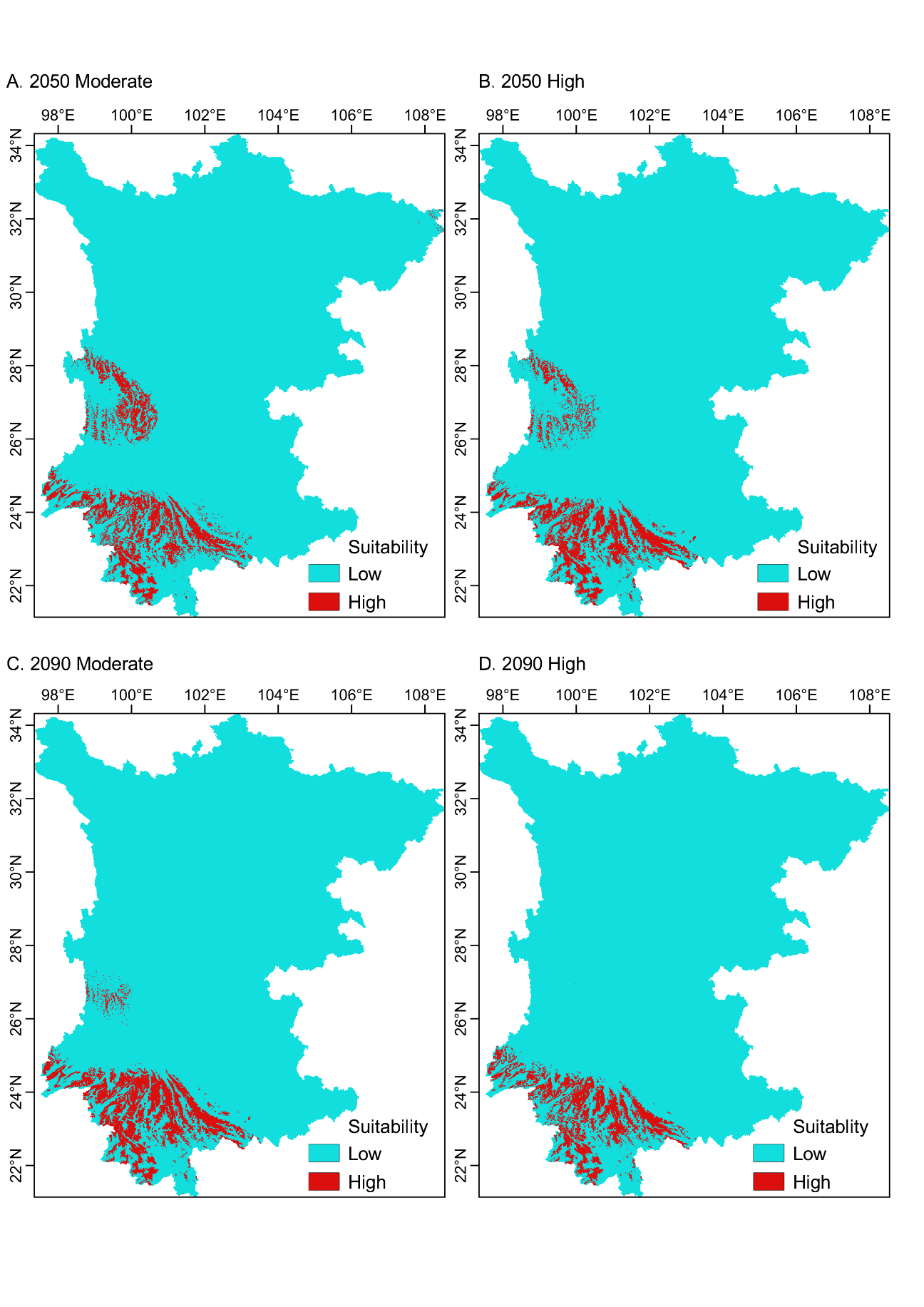


**Additional file 1: Table S1** Average occurrence probability of *L. scutellare* in the cities of Yunnan and Sichuan Provinces, and the numbers of case reports of HFRS and Scrub typhus in Yunnan

|  | Province | City | HFRS | Scrub typhus | Average occurrence probability |
| --- | --- | --- | --- | --- | --- |
| 1 | Sichuan | Aba | NA | NA | 0.174 |
| 2 | Sichuan | Chengdu | NA | NA | 0.070 |
| 3 | Sichuan | Deyang | NA | NA | 0.072 |
| 4 | Sichuan | Ganzi | NA | NA | 0.214 |
| 5 | Sichuan | Mianyang | NA | NA | 0.106 |
| 6 | Sichuan | Yaan | NA | NA | 0.160 |
| 7 | Sichuan | Bazhong | NA | NA | 0.078 |
| 8 | Sichuan | Dazhou | NA | NA | 0.165 |
| 9 | Sichuan | Guangyuan | NA | NA | 0.080 |
| 10 | Sichuan | Nanchong | NA | NA | 0.051 |
| 11 | Sichuan | Meishan | NA | NA | 0.072 |
| 12 | Sichuan | Ziyang | NA | NA | 0.050 |
| 13 | Sichuan | Guangan | NA | NA | 0.099 |
| 14 | Sichuan | Suining | NA | NA | 0.050 |
| 15 | Sichuan | Liangshan | NA | NA | 0.279 |
| 18 | Sichuan | Leshan | NA | NA | 0.094 |
| 19 | Sichuan | Yibin | NA | NA | 0.065 |
| 20 | Sichuan | Zigong | NA | NA | 0.052 |
| 22 | Sichuan | Panzhihua | NA | NA | 0.431 |
| 28 | Sichuan | Luzhou | NA | NA | 0.167 |
| 29 | Sichuan | Neijiang | NA | NA | 0.050 |
| 16 | Yunnan | Diqing | 3 | 3 | 0.346 |
| 17 | Yunnan | Nujiang | 8 | 132 | 0.458 |
| 21 | Yunnan | Zhaotong | 13 | 481 | 0.147 |
| 23 | Yunnan | Chuxiong | 251 | 2778 | 0.418 |
| 24 | Yunnan | Dali | 472 | 667 | 0.642 |
| 25 | Yunnan | Kunming | 314 | 630 | 0.413 |
| 26 | Yunnan | Lijiang | 35 | 328 | 0.436 |
| 27 | Yunnan | Qujing | 39 | 63 | 0.236 |
| 30 | Yunnan | Baoshan | 4 | 6534 | 0.424 |
| 31 | Yunnan | Dehong | 2 | 3586 | 0.255 |
| 32 | Yunnan | Lincang | 16 | 5141 | 0.303 |
| 33 | Yunnan | Puer | 2 | 1444 | 0.274 |
| 34 | Yunnan | Yuxi | 12 | 1063 | 0.305 |
| 35 | Yunnan | Honghe | 156 | 2407 | 0.273 |
| 36 | Yunnan | Wenshan | 10 | 384 | 0.239 |
| 37 | Yunnan | Xishuangbanna | 0 | 1208 | 0.174 |
